# Using Theory to Develop *Healthy Choices in Motion*, a Comprehensive, Experiential Physical Activity Curriculum

**DOI:** 10.3389/fpubh.2019.00421

**Published:** 2020-01-23

**Authors:** Deborah S. Fetter, Jessica D. Linnell, Madan Dharmar, Jacqueline J. Bergman, Michele Byrnes, Melanie A. Gerdes, Lyndsey D. Ruiz, Natalie Pang, Jona Pressman, Rachel E. Scherr

**Affiliations:** ^1^Department of Nutrition, University of California, Davis, Davis, CA, United States; ^2^Department of Nutrition, Center for Nutrition in Schools, University of California, Davis, Davis, CA, United States; ^3^Oregon State University Extension Service, Corvallis, OR, United States; ^4^Betty Irene Moore School of Nursing, University of California, Davis, Davis, CA, United States; ^5^St. Mary's College of California, Allied Health Sciences Program, Moraga, CA, United States; ^6^CalFresh Healthy Living, University of California, Davis, Davis, CA, United States; ^7^Butte County Cluster, University of California Agriculture and Natural Resources, Oroville, CA, United States

**Keywords:** physical activity, experiential learning, social cognitive theory, shaping healthy choices program, curriculum

## Abstract

**Background:** Research has shown that engaging in regular physical activity supports physiologic, metabolic, and immunologic processes, as well as quality of life. However, few youth in the United States meet the U.S. Department of Health and Human Services recommendation of 60 min of moderate-to-vigorous physical activity every day. School-based programs can be an effective avenue for engaging youth in physical activity, particularly when the design of the health education is based on theory, research, and practice. The purpose of this study was to design, implement, and evaluate a theory-driven physical activity curriculum for the Shaping Healthy Choices Program (SHCP) using a systematic approach.

**Methods:** The experiential, inquiry-based physical activity curriculum, Healthy Choices in Motion (HCIM), was developed with an optional technology enhancement using Backward Design. A questionnaire to assess the curriculum's effect on physical activity knowledge was developed and assessed for content validity, internal consistency (α = 0.84), and test-retest reliability (r = 0.73). The curriculum was piloted in two phases among upper elementary-aged youth: to ensure the learning goals were met (Pilot I) and to determine the curriculum's impact on physical activity knowledge, behavior, and self-efficacy (Pilot II). Pilot II was implemented among eight 4th and 5th-grade classrooms participating in the UC CalFresh Nutrition Education Program: (1) Comparison (no intervention) (*n* = 25); (2) SHCP only (*n* = 22); (3) SHCP + HCIM (*n* = 42); (4) SHCP + HCIM with technology enhancement (*n* = 47). Analyses included unadjusted ANOVA and Bonferroni for multiple comparisons and paired *t*-test (*p* < 0.05).

**Results:** Through the use of a methodical design approach, a comprehensive physical activity curriculum, called HCIM, was developed. Youth participating in HCIM improved physical activity knowledge compared to youth receiving no intervention (+2.8 points, *p* = 0.009) and youth only in the SHCP (+3.0 points, *p* = 0.007). Youth participating in HCIM with technology enhancement demonstrated improvements compared to youth only in the SHCP (+2.3 points, *p* = 0.05).

**Conclusion:** Improvements in physical activity knowledge in youth participating in HCIM may contribute to improvements in physical activity and should be further explored in conjunction with behavioral measurements.

## Introduction

Obesity is a multifactorial health condition, thus employing a single approach for obesity-prevention is unlikely to succeed (1). Research has shown that regular physical activity (PA) helps with weight control and improves physiologic, metabolic, and immunologic processes, along with quality of life (2). However, the proportion of time youth spend in sedentary activity continues to increase and few meet the U.S. Department of Health and Human Services recommendation of 60 min of moderate-to-vigorous physical activity (MVPA) every day (3, 4). Physical activity is a desirable, modifiable factor to target in obesity-prevention programs because it is linked to improved body composition and other determinants of health (3). Engaging youth in PA is postulated to be an effective obesity-prevention strategy since being active in childhood is associated with a greater likelihood of being active throughout life (5). Previous obesity-prevention programs that have a focus on PA education, such as the Coordinated Approach to Child Health (CATCH) program and Sports, Play & Active Recreation for Kids (SPARK) program, have shown promising results related to increasing MVPA and improving PA patterns (6, 7). While these programs have produced exciting results, overall there remains a lack of time allocation for PA promotion in the school setting (8, 9).

To address the increasing prevalence of childhood obesity, a school-based, multicomponent nutrition intervention was developed, called the Shaping Healthy Choices Program (SHCP) (10). The SHCP was evaluated in 4th-grade youth during the 2012–2013 school year. Although improvements in PA intensity were observed during the initial pilot study (11, 12), SHCP educators have reported a growing need for PA education in schools, especially as physical education programs continue to be eliminated (13, 14). Further, research suggests that the limited physical education in the school setting may not be enough to encourage youth to be physically active (7, 15). Incorporating a comprehensive PA curriculum into the SHCP would not only allow for the structured time to perform activities, but also provide youth with background knowledge about the importance of PA (13).

In 2014, the SHCP researchers partnered with the University of California CalFresh Nutrition Education Program (UC CalFresh), one of the Supplemental Nutrition Assistance Program Education (SNAP-Ed)-implementing agencies in California, to disseminate the SHCP in multiple counties across California. In partnership with UC CalFresh and funded by University of California Agriculture and Natural Resources (UC ANR), the SHCP researchers designed a complementary PA curriculum that addresses a combination of cultural, environmental, and knowledge factors, which have been shown to influence the likelihood of youth engaging in PA (2). Although, effective PA curricula exist, utilizing innovative approaches in curricula, such as experiential learning, inquiry-based education, and technology, could help support future interventions and encourage sustainability of the program in educational settings (16). Research needs to be conducted to investigate how incorporating these features into a PA curriculum affects attainment of PA knowledge. Detailing the development of the novel activities within this curriculum could help other researchers replicate the intervention in additional settings (15). Furthermore, few school-based interventions utilize wearable activity devices for youth, as technology-related interventions are a relatively new area for obesity-prevention research (17). Previous research has demonstrated that wearable activity monitoring devices can be effective in providing motivation to engage in PA, thus these devices may become a critical component of successful behavioral interventions (17). The purpose of this paper is to describe the systematic development process and pilot testing for a comprehensive PA curriculum and PA knowledge questionnaire.

## Materials Methods

### Curriculum Development and Pilot I

A curriculum was developed to provide youth with opportunities to learn about the importance and enjoyment of PA. The development team was convened in 2015 to design this curriculum using the methodology from the development of the nutrition curriculum from the SHCP (18). This team was made up of five nutrition researchers (two nutrition faculty, two postdoctoral scholars, and one doctoral candidate), one PA expert, two nutrition education program staff, and one undergraduate nutrition student. The team had expertise in the areas of nutrition science, nutrition and PA education, kinesiology, psychology, inquiry-based education, and curriculum development. The team ensured the modules met the curriculum objectives and aligned with educational standards across multiple subjects. The modules were grounded in theoretical constructs and concepts were layered using a technique called scaffolding to improve PA knowledge and skills. Scaffolding refers to using educational techniques to progressively guide youth toward a greater understanding of the material (19).

The Social Cognitive Theory (SCT) (20) and constructivism (21) were the theoretical frameworks for curriculum activities. Behavioral capability, reciprocal determinism, and self-efficacy were identified as the primary SCT constructs utilized. The pedagogical strategy for the curriculum was experiential learning through guided inquiry (22). Relying upon the principles of the SCT (20) and constructivism (21), the backward design approach (23) was used to develop the curriculum. This process involves three steps: (1) establish learning outcomes; (2) ascertain acceptable evidence of learning; and (3) create activities to align with learning outcomes and evidence of learning (22).

To establish learning outcomes, the development team reviewed national and state physical education and health education standards, Common Core State Standards for Mathematics and English Language Arts, Next Generation Science Standards, and MyPlate recommendations. The team determined that acceptable evidence of learning would be demonstrated through completing worksheets or verbalizing specific words/phrases. The final step was to create activities that aligned with the identified learning outcomes and allowed for youth to demonstrate evidence of learning. The module activities used guided inquiry organized around the five-step Experiential Learning Cycle, as described by Kolb and modified by Pfeiffer and Jones (22). This cycle involves a sequence of phases in which learners engage in a learning experience (Experience), share their findings (Sharing), process their experience through reflection (Processing), generalize their experience to real-world examples (Generalizing), and then finally apply what they have learned in new contexts (Apply) (22). Open-ended prompts were embedded in the procedure to encourage youth to articulate their thinking processes.

The module activities were designed around foundational knowledge and skills to advance behavioral capability with scaffolding concepts. The team built in opportunities to enhance self-efficacy around being active by including structured journal activities, in which youth would expand on concepts learned in the modules, monitor their activity, and set goals. Youth recorded the time spent doing PA and created bar graphs to visualize their progress.

The development team met weekly for seven months during the 2015–2016 academic year. During this time, a two-phase pilot test (Pilot I) was conducted in after-school programs in Davis, CA and Woodland, CA, respectively. The objective of the first phase of Pilot I was to determine whether the intended learning concepts were achieved and the activity designs were appropriate for an upper-elementary age group. To accomplish this, the team facilitated the modules with youth at the pilot site and engaged in reflective practice afterwards using the observations made during the lesson. After the discussion, subsequent revisions were made, as needed. Observations were recorded during the facilitation using a plus/delta tool to provide qualitative information about strengths of the lesson (plus) and areas for improvement (delta). Pedometers were used as a technology enhancement during the first pilot phase. Following revisions, the curriculum was tested again for phase two of Pilot I in a different after-school program in Woodland, CA in order to facilitate the modules with a population of low-income, diverse youth, similar to the intended target group. In this phase, the curriculum was facilitated by one researcher while two members of the research team recorded observations using the plus/delta tool for further curriculum refinement. Accelerometers (TupeloLife, Dallas, TX) were used as a technology enhancement in this phase, and youth were able to log into their accounts to view their data and record the information in their journals. The University of California, Davis Human Subjects Institutional Review Board approved the curriculum development process as an exempt protocol.

### Physical Activity Knowledge Questionnaire Development

A 20-item questionnaire to assess PA knowledge was developed in conjunction with the curriculum with the purpose of providing a reliable evaluation tool for the current study and future studies involving the curriculum. Using the primary learning concepts, the questions were designed to align with the key concepts emphasized in the modules. Nutrition and PA education experts reviewed the questionnaire for content validity. To assess test-retest reliability and internal consistency, the questionnaire was administered at two time-points with 1 week in between to youth who attended the after-school program in phase two of Pilot I.

Analyses were conducted using Stata 14 software (StataCorp, College Station, TX, 2015). Pearson's correlations were used to assess test-retest reliability by comparing scores between the test and retest administration with significance set at *p* ≤ 0.05. Cronbach's alpha was used to determine internal consistency, with a minimum acceptable level of α > 0.70.

### Pilot Study II

During spring 2016, following Pilot I from the curriculum development phase, eight 4th- and 5th-grade classrooms (across four schools) were selected to participate in a 7-week quasi-experimental pilot study (Pilot II) to evaluate the effect of the PA curriculum on PA knowledge. These classrooms were already participating in the UC CalFresh Nutrition Education Program in Butte County. At one school, two classrooms not participating in the SHCP were selected to serve as the comparison group. Six of the classrooms (across three schools) were selected because of their current participation in the SHCP. Of these, two classrooms served as a SHCP-only comparison group, two classrooms were selected to participate in the PA curriculum, and two classrooms were selected to participate in the PA curriculum with the technology enhancement. The SHCP-only group was selected to determine that any changes in outcomes were attributed to the curriculum and not to other components of the SHCP.

### Pilot Study II Participant Recruitment

Recruitment using an active informed consent process took place over 2 weeks using a modified Dillman method (24). Youth were sent home with a packet that included a flier, a youth information letter, a parent letter, a consent form, and a demographics questionnaire. Both English and Spanish versions of these documents were provided. Each classroom was given a poster board promoting the goal of at least 80% of the class returning the consent forms, regardless of study participation permission (25). The University of California, Davis Human Subjects Institutional Review Board reviewed and approved all procedures for human research and determined that this study protocol was expedited.

### Pilot Study II Data Collection

Data were collected as pre- and post-measures by trained researchers. Family demographics were collected through the questionnaire sent home and returned to the teachers. Prior to facilitating the PA curriculum, the research team collected baseline data, which included height/weight and PA knowledge, behavior, and self-efficacy. Physical activity behavior was measured by the Physical Activity Questionnaire for Children (PAQ-C) (26). Youth completed the Active Winners Psychosocial Scales Constructs questionnaire to measure PA self-efficacy (27). Accelerometers (TupeloLife, Dallas, TX) were distributed to all participating youth. As per the design of the clip-on accelerometer, youth were instructed to wear the accelerometer clipped to their pants' pocket at all times during the 7-week pilot study (Pilot II). While research has shown that compliance increases with wrist-worn accelerometers, the accuracy decreases as compared to accelerometers that are worn toward the middle of the body (28). Follow-up data collection for all groups occurred 1 week after the last lesson was delivered.

### Pilot Study II Curriculum Facilitation

The PA curriculum was administered over 5 weeks where each module was facilitated once a week for 40–60 min in four classrooms. Two classrooms received the curriculum with the technology enhancement and youth were able to view their data through a web-based portal to record the number of steps and active minutes from the accelerometer in their journals.

### Pilot Study II Statistical Analysis

Analyses were conducted on data from youth that completed both pre- and post-assessments. For all outcomes, means and standard deviations (SDs) for each group were calculated and distributions were examined for normality using histograms, skewness, and Kurtosis. Changes in outcomes were calculated by subtracting pre-scores from post-scores. Physical activity behavior scores were calculated following the scoring procedure in the PAQ-C manual (26). The self-efficacy questionnaire was divided into construct sections for beliefs, social support, and self-efficacy and analyzed by section. Descriptive statistics were expressed as means and SDs for continuous variables and percentages for categorical variables. Baseline characteristics were compared between the groups. Categorical variables were calculated into percentages and groups were compared using the chi-square test for homogeneity or Fisher's exact test, as appropriate. As this was a pilot study with a small sample, unadjusted ANOVA and Bonferroni for multiple comparisons were used to compare differences in changes in scores between pre- and post- measurements for PA knowledge and related characteristics. Paired *t*-tests were used for pre- and post- comparisons within groups. Stata 14 software (StataCorp, College Station, TX, 2015) was used for all statistical analyses. Statistical significance was determined using *p* ≤ 0.05.

## Results

### Curriculum Development and Pilot I

Through the process of backward design, the development team identified learning outcomes for the curriculum and corresponding evidence of learning (Table 1). Suitable evidence of learning for each module was determined by identifying how youth could showcase understanding of learning objectives.

**Table 1:** Identification of learning outcomes and evidence of learning.

**Module sequence**	**Concept**	**Determination of learning (students will be able to…)**
1	Benefits of PA	• Describe how it feels to engage in PA • Compare how their body responded before, during, and after physical activity • Identify at least three benefits of PA
2	PA recommendations across the lifespan	• Add up minutes of PA to see if PA recommendations were met • Compare PA recommendations across the lifespan • Identify PA intensity levels
3	The five components of physical fitness (flexibility, muscular endurance, cardiovascular endurance, muscular strength, and body composition)	• Categorize activities into the five components of physical fitness • Understand how their body responded during physical activity
4	Variety of PA to achieve fitness	• Identify non-traditional PA
5	All of the concepts “bringing it all together”	• Synthesize the concepts from all the lessons
All	PA is fun	• Identify a PA they enjoy • Set a personal PA goal in their journal

The sequential module activities were formulated from the learning objectives with the goal of improving knowledge and reasoning skills related to PA (Table 2). The purpose of these activities was for youth to acquire behavioral capability through constructing foundational knowledge and skills. The results of the structured observational data collected during the pilot test were used to refine the procedures and activities, as necessary. Upon completion of Pilot I, the *Healthy Choices in Motion* (HCIM) curriculum was finalized and consisted of five, 40–60-min classroom activities and the structured journal.

**Table 2:** Summary of *Healthy Choices in motion* activities.

	**Module**	**Application of social cognitive theory**	**Classroom activity**	**Journal activity**
1	The benefits of PA	*Behavioral capability:* develop an understanding of the benefits of PA *Reciprocal determinism:* journal activity to encourage family members to be active together *Self-efficacy:* set PA goals for the next week	Investigate how PA affects the body	Track, chart, and reflect on weekly PA; identify health benefits
2	PA recommendations	*Behavioral capability:* understand the differences between intensity of PA and how it relates to health benefits and the recommendations; acquire knowledge to understand and apply PA recommendations *Reciprocal determinism:* journal activity to encourage family members to meet PA recommendations *Self-efficacy:* set PA goals for the next week to meet PA recommendation	Explore the PA intensity levels and identify PA recommendations	Track, chart, and reflect on weekly PA; identify the intensity
3	Components of physical fitness	*Behavioral capability:* develop knowledge and skills in the different components of physical fitness *Self-efficacy:* set PA goals for the next week to include more components of physical fitness	Explore the five components of physical fitness	Track, chart, and reflect on weekly PA; categorize PA into the components of physical fitness
4	We need a variety of PA	*Behavioral capability:* generate interest in different types of PA *Reciprocal determinism:* journal activity to encourage family members to engage in non-traditional PA together *Self-efficacy:* set PA goals for the next week to include non-traditional PA	Identify non-traditional PA	Track, chart, and reflect on weekly PA; identify non-traditional PA
5	Being a physically active person	*Behavioral capability:* understand there are many ways to be a physically active person *Self-efficacy:* set short- and long-term PA goals	Visually depict what being physically active means to the student	Reflect on the learning objectives and set PA goals

Nutrition and PA education experts determined that the PA knowledge questionnaire had acceptable content validity. Reliability testing of the questionnaire demonstrated acceptable test-retest reliability (r = 0.73) and internal consistency (α = 0.84).

### Pilot II

Youth were recruited and divided by classroom into one of four groups: Comparison (*n* = 25), SHCP-only (*n* = 22), HCIM (*n* = 42), and HCIM with technology enhancement (*n* = 47). Baseline characteristics are presented in Table 3.

**Table 3:** Characteristics of youth participating in Pilot II.

**Characteristics**		**Comparison (no intervention) (*n* = 25)**	**SHCP (*n* = 22)**	**SHCP + HCIM (*n* = 42)**	**SHCP + HCIM w/tech (*n* = 47)**	***p***
Sex, *n* (%)	Female	14 (56)	12 (57)	22 (56)	26 (59)	0.1
Unreported		0 (0)	1 (5)	3 (7)	3 (6)	
Age in years, mean (SD)		10.4 (0.8)	10.5 (0.5)	9.5 (0.6)	10.0 (0.9)	<0.001
Ethnicity/race, *n* (%)						<0.001
	Caucasian/White, not of Hispanic origin	2 (8)	8 (36)	25 (60)	20 (42)	
	Latino/Hispanic (Mexican-American, Puerto Rican, Cuban, Chicano)	21 (84)	7 (32)	6 (14)	6 (13)	
	Other^a^	2 (8)	6 (27)	9 (21)	16 (34)	
	Unreported^b^	0 (0)	1 (5)	2 (5)	5 (11)	
Income, *n* (%)						0.11
	$0–$19,999	7 (28)	1 (5)	10 (24)	4 (9)	
	$20,000–$39,999	10 (40)	6 (29)	12 (28)	8 (17)	
	$40,000–$59,999	3 (12)	5 (23)	2 (5)	6 (13)	
	$60,000–$79,999	2 (8)	2 (10)	6 (14)	7 (15)	
	$80,000–$99,999	0 (0)	2 (10)	3 (7)	2 (4)	
	$100,000 or more	1 (4)	5 (23)	4 (10)	10 (21)	
	Unreported^b^	2 (8)	0 (0)	5 (12)	10 (21)	
Highest education completed by the household, *n* (%)						0.13
	<8th grade, 8th−11th grade, finished high school or have GED	22 (88)	13 (59)	25 (60)	26 (55)	
	Vocational or technical training, or some college	0 (0)	2 (9)	1 (2)	2 (4)	
	Associate's degree, Bachelor's degree, or Postgraduate	2 (8)	6 (27)	15 (36)	12 (26)	
	Unreported^b^	1 (4)	1 (5)	1 (2)	7 (15)	
Body mass index (kg/m^2^; *n*, %)						0.28
	Underweight	1 (4)	0 (0)	0 (0)	2 (4)	
	Normal weight	7 (28)	13 (62)	23 (55)	25 (53)	
	Overweight	8 (32)	5 (24)	8 (19)	10 (21)	
	Obese	9 (36)	3 (14)	8 (19)	7 (15)	
	Unreported^b^	0 (0)	0 (0)	3 (7)	3 (6)	

a *Includes African American/Black, American Indian/Alaska Native, Asian/Pacific Islander, and multiple reported*.

b *Unreported includes those that did not return the questionnaire, those that left the question blank, and students that were absent during height and weight collection*.

Table 4 shows the comparison of individual outcomes pre- and post-intervention. Youth that participated in HCIM demonstrated a 14% greater improvement in PA knowledge compared to the comparison group (difference of 2.8 points; *p* = 0.009) and a 15% greater improvement compared to the SHCP-only group (difference of 3.0 points; *p* = 0.007). Youth that participated with the technology enhancement also demonstrated a 12% greater improvement in PA knowledge compared to the SHCP-only group (difference of 2.3 points, *p* = 0.05). There were no differences when comparing the technology enhancement to the comparison group and HCIM group.

**Table 4:** Comparison of individual outcomes pre-measure and post-measure.

**Measures**	**Comparison (no intervention)**	**SHCP**	**SHCP + HCIM**	**SHCP + HCIM w/tech**
**PHYSICAL ACTIVITY KNOWLEDGE SCORE^1^ [MEAN (SD)]**				
*n*	23	19	36	40
Pre^†^	13.3 (2.3)	13.6 (2.0)	10.1 (3.4)	12.2 (2.8)
Post	13.5 (2.3)	13.5 (2.4)	12.9 (3.3)	14.5 (4.0)
Change^*^	0.13^ab^ (2.6)	−0.1^a^ (2.4)	2.9^c^ (3.4)	2.3^bc^ (3.6)
**PHYSICAL ACTIVITY BEHAVIOR COMPOSITE SCORE^2^ [MEAN (SD)]**				
*n*	24	20	40	44
Pre	3.7 (0.6)	3.1 (0.8)	3.3 (0.8)	3.4 (0.8)
Post	3.6 (1.0)	2.8 (1.0)	3.0 (1.2)	3.3 (1.3)
Change	−0.02 (0.7)	−0.2 (0.8)	−0.3 (0.9)	−0.1 (1.1)
**PSYCHOSOCIAL DETERMINANTS OF PHYSICAL ACTIVITY: BELIEFS^3^ [MEAN (SD)]**				
*n*	23	14	37	40
Pre	11.35 (1.87)	10.71 (2.13)	11.7 (1.75)	11.38 (2.08)
Post	11.35 (1.61)	10.93 (2.2)	11.14 (2.47)	10.95 (2.32)
Change	0 (1.86)	0.21 (1.53)	−0.57 (1.8)	−0.43 (2.8)
**PSYCHOSOCIAL DETERMINANTS OF PHYSICAL ACTIVITY: SOCIAL SUPPORT^4^ [MEAN (SD)]**				
*n*	23	14	37	40
Pre	5.57 (1.88)	5.64 (2.37)	4.97 (2.37)	5.22 (2.24)
Post^*^	6.17 (1.67)	5.93 (2.09)	4.68 (2.12)	6.1 (2.16)
Change^*^	0.61 (1.59)	0.29 (1.32)	−0.30^a^ (2.18)	0.88^b^ (1.91)
**PSYCHOSOCIAL DETERMINANTS OF PHYSICAL ACTIVITY: SELF-EFFICACY^5^ [MEAN (SD)]**				
*n*	23	14	37	41
Pre	14.48 (2.48)	14.36 (1.64)	13.3 (4.03)	14.22 (2.83)
Post	15 (1.93)	14.21 (2.36)	12.54 (4.04)	14.12 (3.2)
Change	0.52 (2.63)	−0.14 (1.4)	−0.76 (2.66)	−0.1 (2.48)
**BMI PERCENTILE [MEAN (SD)]**				
*n*	23	21	38	41
Pre	80.0 (29.4)	70.6 (23.5)	65.8 (30.5)	64.0 (30.0)
Post	78.8 (30.9)	68.8 (24.3)	64.8 (30.8)	64.8 (29.1)
Change	−1.2 (2.0)	−1.8 (3.8)	−1.1 (4.4)	0.8 (4.6)

*Values with different alphabetical superscripts denote significance of P < 0.05*.

* *Global F-test is significant*.

† *Baseline differences between groups are indicated*.

1 *Minimum score = 0; Maximum score = 20*.

2 *Minimum score = 1; Maximum score = 5*.

3 *Maximum score = 15*.

4 *Maximum score = 8*.

5 *Maximum score = 17*.

Youth that participated in HCIM with technology enhancement demonstrated significant improvements in their perceived ability to seek social support to help them be physically active as compared to the HCIM group (difference of 1.2 points; *p* = 0.045). There were no additional differences between the groups. All additional analyses resulted in null findings (data not shown). While youth were instructed to wear the accelerometers for the duration of the study, the low compliance prevented analyses on these data from being conducted.

## Discussion

Through the use of a methodical design approach, a comprehensive PA curriculum, called *Healthy Choices in Motion*, was developed with measurable learning outcomes. Using a process grounded in education theory and applying this level of rigor to curriculum development can help strengthen future health education research by ensuring learning outcomes are achieved and can be evaluated (18).

*Healthy Choices in Motion* can provide a structured, interactive learning environment in which youth can be active and learn. Based on the SCT, behavior change is influenced by reciprocal determinism between the individual, environment, and behavior (20). *Healthy Choices in Motion* aims to improve knowledge about PA as an individual-level factor which helps prepare youth to make a change. *Healthy Choices in Motion* also provides opportunities for reflection and goal-setting to improve perceived self-efficacy, as this relates to behavioral capability. Research has also shown that perceived self-efficacy is a positively correlated psychosocial determinant of PA (2). Behavior change is partly impacted by the resulting social reactions, thus the team-oriented approach to HCIM encourages social support for being physically active, which may help influence the cultural norms and practices of the classroom through behavioral modeling (29).

Pilot II was employed to evaluate the impact of the curriculum on PA knowledge using the questionnaire designed during the curriculum development. After participating in the HCIM curriculum, youth improved PA knowledge, regardless of the technology enhancement. This shows that future educators using HCIM can achieve the same learning goals with or without the technology enhancement.

In Pilot II, determinants of PA were also assessed. Youth did not report a change in PA behavior from pre- to post-. However, the SCT postulates that knowledge precedes behavior change, so the youth who participated in HCIM may need a longer study duration to achieve behavior change (20). There were no differences in beliefs about PA or self-efficacy among the groups, which is consistent with other community-oriented PA programs for this age group and study duration (13). Interestingly, youth that participated in HCIM with technology enhancement reported significant improvements in seeking social support to be physically active. The routine of recording data from the accelerometers may have been an added motivation and could have encouraged youth to discuss their data with each other (17, 30). Youth that participated only in HCIM did not exhibit these improvements, which could mean there may have been something unique about the classroom environment between groups that contributed to these differences.

Although many programs have been effective at engaging youth in PA (6, 7), HCIM provides an approach to PA education that utilizes guided-inquiry pedagogy, incorporates technology, and aligns with educational standards across subjects while providing youth with knowledge, experience, and social support. Further, the curriculum focuses on learning about the enjoyment of PA and addressing other determinants of health. This curriculum also encourages youth to replace sedentary activity with light intensity activity due to the associated health benefits (28).

The experiential and inquiry-based structure of HCIM encourages youth to think critically. Experiential learning is a theoretical perspective that emphasizes the experiences of the learners and focuses on the learning process so that ideas are formed and re-formed as youth experience the activity (22). Developing curricula based on the experiential learning perspective may promote beneficial outcomes by engaging youth through educational experiences (31).

In conjunction with HCIM, a questionnaire to measure PA knowledge was developed and assessed for content validity, internal consistency, and test-retest reliability. Performing this level of analysis on questionnaire development helps confirm the questionnaire is reliable for subsequent use in studies.

There were several limitations to Pilot II. Convenience sampling was employed and the sample size was relatively small, which resulted in differences in baseline characteristics for age and race/ethnicity distribution. Pilot II was also conducted over a short period (7-weeks), thus a longer duration may be needed to see improvements in other determinants of PA (32). Self-report questionnaires were used to collect information about these other determinants of PA, yet these are known to have limitations due to recall bias and response bias. Because of the self-report methodology, youth may have overestimated the amount of time they were being active and been subject to social desirability (33). Future studies should consider using an objective measure of PA, such as accelerometers designed for youth, in combination with a questionnaire. This combination would be useful since accelerometers provide a fairly accurate measurement of activity, but many devices are unable to measure certain activities, such as swimming, which can instead be captured through questionnaires (17). Additionally, choosing an accelerometer designed with youth preferences and more interactive features would increase the compliance toward wearing the device (12).

One consideration for implementing health education programs in schools is the limited classroom time (15, 34). To address this, HCIM was designed to align with current education standards across subjects to meet the needs of teachers, while also providing an opportunity for youth to explore PA. Developers of future health education programs may consider aligning curricula with current standards as a way to incentivize schools to adopt the program (34, 35).

## Conclusions

The impetus for designing HCIM was to strengthen the SHCP by adding a discrete PA component that provides youth with background for why PA is important for health. While there are several excellent resources available, the emphasis on deepening PA knowledge complementary to engaging in PA is what makes HCIM a unique educational experience. Additionally, this comprehensive, theory-based PA curriculum was created using a systematic approach to curriculum development that provided a strong framework for methodological design. This process enables researchers to design health education curricula with measurable learning outcomes that can be evaluated during subsequent research studies. Further, the reflective practice during the piloting phases allows for curriculum activities to be tested and refined before conducting a subsequent study. The documented improvements in PA knowledge in youth that participated in HCIM with or without the technology enhancement demonstrates that this PA curriculum improves PA knowledge. According to the SCT, there is a link between knowledge and behavior, thus this improvement in PA knowledge provides the framework for youth to improve PA patterns over time (20). Future research is needed to determine if youth participating in HCIM continue to improve PA knowledge, which could contribute to improvements in PA-related behaviors. For subsequent SHCP implementation, the inclusion of HCIM may add to the improvement observed in PA intensity patterns from the initial SHCP pilot intervention (12).

## Data Availability Statement

The datasets generated for this study are available on request to the corresponding author.

## Ethics Statement

The studies involving human participants were reviewed and approved by University of California, Davis Institutional Review Board. Written informed consent to participate in this study was provided by the participants' legal guardian/next of kin.

## Author Contributions

DF led the study implementation (curriculum development, Pilot I, Pilot II), collected and analyzed the data, and wrote the manuscript. RS, JL, JB, MB, MG, LR, and NP formed the curriculum development team and assisted with Pilot I. DF, RS, and JL developed and tested the PA questionnaire for test-retest reliability, internal consistency, and content validity. JP assisted with recruiting the classrooms for Pilot II. MD assisted with data analysis from Pilot II. All authors read and reviewed the manuscript.

### Conflict of Interest

The authors declare that the research was conducted in the absence of any commercial or financial relationships that could be construed as a potential conflict of interest.
